# Redetermination of 1-carboxy­cyclo­hexan-1-aminium chloride

**DOI:** 10.1107/S1600536808044243

**Published:** 2009-01-08

**Authors:** Lusbely M. Belandria, Gerzon E. Delgado, Asiloé J. Mora, Luis E. Seijas, Teresa González

**Affiliations:** a Laboratorio de Cristalografía, Departamento de Química, Facultad de Ciencias, Universidad de Los Andes, Mérida 5101, Venezuela; b Centro de Química, Instituto Venezolano de Investigaciones Científicas (IVIC), Apartado 21827, Caracas 1020-A, Venezuela

## Abstract

The crystal structure of the title compound, C_7_H_14_NO_2_^+^·Cl^−^, was reported previously [Chacko, Srinivasan & Zand (1975 ▶). *J. Cryst. Mol. Struct.* **5**, 353–357] from Weissenberg photographic data with *R* = 0.113. It has now been redetermined, providing a significant increase in the precision of the derived geometric parameters, *viz*. mean σ(C—C) = 0.003 Å in the present work compared with 0.021 Å for the previous work. The complete cation is generated by crystallographic mirrror symmetry, with three C atoms, two O atoms and the N atom lying on the reflecting plane; the chloride anion also has *m* site symmetry. The crystal structure is established by a two-dimensional network of O—H⋯Cl and N—H⋯Cl hydrogen bonds, generating *C* ^1^_2_(4) and *C* ^1^_2_(7) chains, and *R* ^2^_4_(8) and *R* ^2^_4_(14) rings.

## Related literature

For the earlier structure determination of the title salt, see: Chacko *et al.* (1971 ▶, 1975 ▶). For related literature, see Rodríguez-Ropero *et al.* (2008 ▶). For the crystal structure of the pure amino acid, see: Valle *et al.* (1988 ▶). For ring conformation analysis, see: Cremer & Pople (1975 ▶). For hydrogen-bond motifs in graph-set notation, see: Etter (1990 ▶).
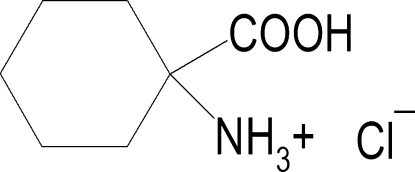


## Experimental

### 

#### Crystal data

C_7_H_14_NO_2_^+^·Cl^−^
*M* *_r_* = 179.64
Monoclinic, 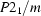

*a* = 7.382 (3) Å
*b* = 6.357 (2) Å
*c* = 9.374 (3) Å
β = 96.239 (10)°
*V* = 437.3 (3) Å^3^
*Z* = 2
Mo *K*α radiation
μ = 0.39 mm^−1^
*T* = 298 (2) K
0.31 × 0.27 × 0.18 mm

#### Data collection

Rigaku AFC-7S Mercury diffractometer
Absorption correction: multi-scan (Jacobson, 1998 ▶) *T*_min_ = 0.880, *T*_max_ = 0.930
4638 measured reflections
845 independent reflections
789 reflections with *I* > 2σ(*I*)
*R*_int_ = 0.023

#### Refinement

*R*[*F* ^2^ > 2σ(*F* ^2^)] = 0.033
*wR*(*F* ^2^) = 0.088
*S* = 1.01
845 reflections
85 parameters
H atoms treated by a mixture of independent and constrained refinement
Δρ_max_ = 0.18 e Å^−3^
Δρ_min_ = −0.15 e Å^−3^

### 

Data collection: *CrystalClear* (Rigaku, 2002 ▶); cell refinement: *CrystalClear*; data reduction: *CrystalStructure* (Rigaku/MSC, 2004 ▶); program(s) used to solve structure: *SHELXS97* (Sheldrick, 2008 ▶); program(s) used to refine structure: *SHELXL97* (Sheldrick, 2008 ▶); molecular graphics: *DIAMOND* (Brandenburg, 1999 ▶); software used to prepare material for publication: *PLATON* (Spek, 2003 ▶) and *publCIF* (Westrip, 2009 ▶).

## Supplementary Material

Crystal structure: contains datablocks I, global. DOI: 10.1107/S1600536808044243/bh2212sup1.cif

Structure factors: contains datablocks I. DOI: 10.1107/S1600536808044243/bh2212Isup2.hkl

Additional supplementary materials:  crystallographic information; 3D view; checkCIF report

## Figures and Tables

**Table 1:** Hydrogen-bond geometry (Å, °)

*D*—H⋯*A*	*D*—H	H⋯*A*	*D*⋯*A*	*D*—H⋯*A*
N1—H1*B*⋯Cl	0.90 (3)	2.34 (3)	3.196 (2)	158 (2)
N1—H1*A*⋯Cl^i^	0.88 (2)	2.58 (2)	3.3816 (13)	152.4 (17)
O1—H1⋯Cl^ii^	0.89 (4)	2.15 (4)	3.027 (2)	168 (3)

Symmetry codes: (i) 
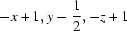
; (ii) 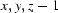.

**Fig. 1.**
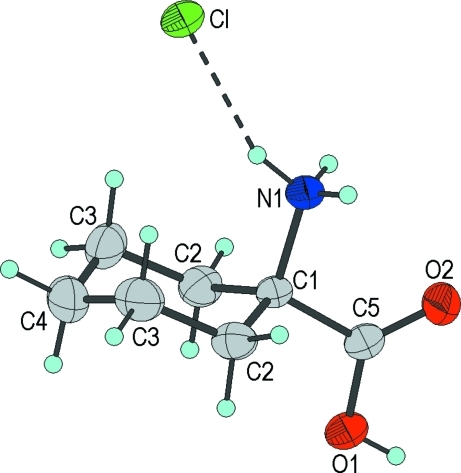
The molecular structure of (I), with atom labels and 50% probability displacement ellipsoids for non-H atoms.

**Fig. 2.**
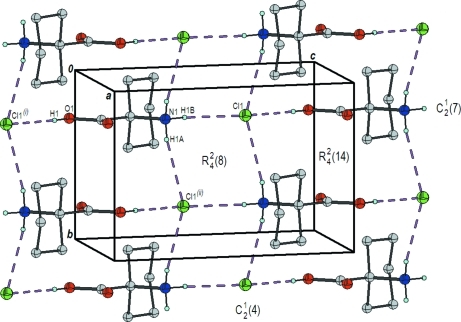
A partial packing view of (I). Hydrogen bonds are shown as dashed lines. H atoms not involved in hydrogen bonding have been omitted for clarity. Symmetry codes: (i) x, y, z - 1; (ii) 1 - x, y - 1/2, 1 - z.

## supplementary crystallographic information

### Comment

1-Amino-cyclohexanecarboxylic acid is a promising amino acid candidate to serve as basic piece in redesigned protein motifs which constitute the basic modules in synthetic nanoconstructs (Rodríguez-Ropero *et al.*, 2008). It structure was reported by Valle *et al.* (1988). The title compound, (I), 1-amino-cyclohexanecarboxylic acid hydrochloride, C_7_H_14_NO_2_^+^.Cl^-^, was first reported in the noncentric space group *P*2_1_ (Chacko *et al.*, 1971) and later reported in the centrosymmetric space group *P*2_1_/*m* (Chacko *et al.*, 1975) with *R* = 0.113. The present paper reports a redetermination of the crystal structure of (I), with greater precision and accuracy. Both, the cation and anion are located on a mirror plane, which confirms the space group *P*2_1_/*m* instead of *P*2_1_. In this compound, the cyclohexane ring adopts a chair conformation, with the ammonium and carboxylate groups in axial and equatorial positions, respectively (Cremer & Pople, 1975), while the pure amino acid has an opposite conformation (Valle *et al.*, 1988). In (I), 1-amino-cyclohexanecarboxylic acid is protonated and is linked to the Cl^-^ anion by a O—H···Cl hydrogen bond (Fig. 1, Table 1). The hydrogen bonds O1—H1···Cl1 (*x*, *y*, *z* - 1) and N1—H1B···Cl1 (1 - *x*, *y* - 1/2, 1 - *z*) form infinite chains running along the [001] direction (Fig. 2) and may be described in graph-set notation as *C*^1^_2_(7) (Etter, 1990). The intramolecular hydrogen bonds N1—H1A···Cl1 form infinite chains, with graph-set *C*^1^_2_(4), running along the *b* cell axis. The combination of these interactions produces rings with graph-set *R*^2^_4_(8) and *R*^2^_4_(14), parallel to the *bc* plane (Fig. 2).

### Experimental

1-Amino-cyclohexanecarboxylic acid and hydrochloric acid in equal molar ratio were mixed together with enough water, and heated to a temperature where a clear solution was obtained. Colorless crystals of (I) suitable for X-ray diffraction analysis were grown by slow evaporation of this solution.

### Refinement

All H atoms were located in a difference map and their positions were freely refined, with the *U*_iso_(H) values set at 1.2*U*_eq_(carrier C), 1.5*U*_eq_(carrier O) and 1.5*U*_eq_(carrier N), respectively.

### Crystal data

**Table:**

C_7_H_14_NO_2_^+^·Cl^−^	*F*(000) = 192
*M**_r_* = 179.64	*D*_x_ = 1.364 Mg m^−^^3^
Monoclinic, *P*2_1_/*m*	Mo *K*α radiation, λ = 0.71070 Å
Hall symbol: -P 2yb	Cell parameters from 1650 reflections
*a* = 7.382 (3) Å	θ = 2.2–27.2°
*b* = 6.357 (2) Å	µ = 0.39 mm^−^^1^
*c* = 9.374 (3) Å	*T* = 298 K
β = 96.239 (10)°	Block, colourless
*V* = 437.3 (3) Å^3^	0.31 × 0.27 × 0.18 mm
*Z* = 2	

### Data collection

**Table:**

Rigaku AFC-7S Mercury diffractometer	845 independent reflections
Radiation source: Normal-focus sealed tube	789 reflections with *I* > 2σ(*I*)
graphite	*R*_int_ = 0.023
Detector resolution: 14.6306 pixels mm^-1^	θ_max_ = 25.0°, θ_min_ = 2.2°
ω scans	*h* = −8→8
Absorption correction: multi-scan (Jacobson, 1998)	*k* = −6→7
*T*_min_ = 0.880, *T*_max_ = 0.930	*l* = −11→11
4638 measured reflections	

### Refinement

**Table:**

Refinement on *F*^2^	Secondary atom site location: difference Fourier map
Least-squares matrix: full	Hydrogen site location: inferred from neighbouring sites
*R*[*F*^2^ > 2σ(*F*^2^)] = 0.033	H atoms treated by a mixture of independent and constrained refinement
*wR*(*F*^2^) = 0.088	*w* = 1/[σ^2^(*F*_o_^2^) + (0.047*P*)^2^ + 0.1831*P*] where *P* = (*F*_o_^2^ + 2*F*_c_^2^)/3
*S* = 1.01	(Δ/σ)_max_ < 0.001
845 reflections	Δρ_max_ = 0.18 e Å^−^^3^
85 parameters	Δρ_min_ = −0.15 e Å^−^^3^
0 restraints	Extinction correction: *SHELXL97* (Sheldrick, 2008), Fc^*^=kFc[1+0.001xFc^2^λ^3^/sin(2θ)]^-1/4^
0 constraints	Extinction coefficient: 0.042 (10)
Primary atom site location: structure-invariant direct methods	

### Fractional atomic coordinates and isotropic or equivalent isotropic displacement parameters (Å^2^)

**Table:**

	*x*	*y*	*z*	*U*_iso_*/*U*_eq_	
Cl	0.41343 (8)	0.2500	0.64377 (6)	0.0414 (3)	
O2	0.5446 (2)	0.2500	0.04758 (18)	0.0511 (5)	
O1	0.2629 (3)	0.2500	−0.06850 (18)	0.0528 (5)	
H1	0.324 (5)	0.2500	−0.146 (4)	0.079*	
N1	0.4370 (3)	0.2500	0.3054 (2)	0.0343 (5)	
H1A	0.506 (3)	0.138 (3)	0.300 (2)	0.051*	
H1B	0.398 (4)	0.2500	0.393 (3)	0.051*	
C1	0.2863 (3)	0.2500	0.1837 (2)	0.0292 (5)	
C2	0.1715 (2)	0.4484 (3)	0.19289 (19)	0.0408 (5)	
H2A	0.251 (3)	0.566 (3)	0.1923 (19)	0.049*	
H2B	0.090 (3)	0.452 (3)	0.107 (2)	0.049*	
C3	0.0597 (3)	0.4450 (4)	0.3193 (2)	0.0539 (6)	
H3A	0.140 (3)	0.449 (4)	0.409 (2)	0.065*	
H3B	−0.018 (3)	0.571 (4)	0.315 (2)	0.065*	
C4	−0.0557 (4)	0.2500	0.3201 (3)	0.0626 (9)	
H4A	−0.145 (5)	0.2500	0.232 (4)	0.075*	
H4B	−0.148 (5)	0.2500	0.397 (4)	0.075*	
C5	0.3819 (3)	0.2500	0.0475 (2)	0.0358 (5)	

### Atomic displacement parameters (Å^2^)

**Table:**

	*U*^11^	*U*^22^	*U*^33^	*U*^12^	*U*^13^	*U*^23^
Cl	0.0457 (4)	0.0544 (4)	0.0238 (4)	0.000	0.0025 (2)	0.000
O2	0.0359 (10)	0.0837 (14)	0.0345 (10)	0.000	0.0078 (7)	0.000
O1	0.0443 (11)	0.0932 (15)	0.0205 (9)	0.000	0.0023 (7)	0.000
N1	0.0335 (11)	0.0449 (12)	0.0238 (10)	0.000	−0.0002 (8)	0.000
C1	0.0303 (11)	0.0355 (12)	0.0211 (11)	0.000	−0.0004 (8)	0.000
C2	0.0439 (10)	0.0402 (10)	0.0373 (10)	0.0074 (8)	−0.0004 (8)	0.0024 (7)
C3	0.0497 (11)	0.0707 (14)	0.0412 (10)	0.0221 (10)	0.0052 (9)	−0.0058 (10)
C4	0.0386 (15)	0.105 (3)	0.0449 (17)	0.000	0.0076 (13)	0.000
C5	0.0385 (13)	0.0425 (13)	0.0262 (12)	0.000	0.0025 (10)	0.000

### Geometric parameters (Å, °)

**Table:**

O2—C5	1.201 (3)	C2—C3	1.516 (3)
O1—C5	1.322 (3)	C2—H2A	0.95 (2)
O1—H1	0.89 (4)	C2—H2B	0.95 (2)
N1—C1	1.504 (3)	C3—C4	1.505 (3)
N1—H1A	0.88 (2)	C3—H3A	0.97 (2)
N1—H1B	0.90 (3)	C3—H3B	0.98 (2)
C1—C5	1.524 (3)	C4—H4A	0.99 (3)
C1—C2	1.528 (2)	C4—H4B	1.05 (4)
			
C5—O1—H1	109 (2)	C4—C3—C2	111.85 (19)
C1—N1—H1A	110.2 (13)	C4—C3—H3A	107.9 (13)
C1—N1—H1B	114.0 (18)	C2—C3—H3A	109.9 (12)
H1A—N1—H1B	107.1 (16)	C4—C3—H3B	110.2 (13)
N1—C1—C5	105.28 (18)	C2—C3—H3B	108.4 (13)
N1—C1—C2	109.04 (12)	H3A—C3—H3B	108.5 (17)
C5—C1—C2	110.97 (12)	C3—C4—H4A	108.6 (10)
C3—C2—C1	112.64 (16)	C3—C4—H4B	114.6 (8)
C3—C2—H2A	113.8 (11)	H4A—C4—H4B	99 (3)
C1—C2—H2A	107.7 (12)	O2—C5—O1	125.2 (2)
C3—C2—H2B	108.3 (11)	O2—C5—C1	123.6 (2)
C1—C2—H2B	105.8 (12)	O1—C5—C1	111.2 (2)
H2A—C2—H2B	108.2 (16)		

### Hydrogen-bond geometry (Å, °)

**Table:**

*D*—H···*A*	*D*—H	H···*A*	*D*···*A*	*D*—H···*A*
N1—H1B···Cl	0.90 (3)	2.34 (3)	3.196 (2)	158 (2)
N1—H1A···Cl^i^	0.88 (2)	2.58 (2)	3.3816 (13)	152.4 (17)
O1—H1···Cl^ii^	0.89 (4)	2.15 (4)	3.027 (2)	168 (3)

Symmetry codes: (i) −*x*+1, *y*−1/2, −*z*+1; (ii) *x*, *y*, *z*−1.
